# Helicobacter pylori infection and positive Rome IV criteria for functional dyspepsia in Romanian medical students

**DOI:** 10.25122/jml-2021-0163

**Published:** 2021

**Authors:** Alexandra Loor, Dan-Lucian Dumitrascu, Teodora Surdea-Blaga, Daniel-Corneliu Leucuta, Liliana David

**Affiliations:** 1. 2^nd^ Medical Department, Iuliu Hatieganu University of Medicine and Pharmacy, Cluj-Napoca, Romania; 2. Department of Medical Informatics and Biostatistics, Iuliu Hatieganu University of Medicine and Pharmacy, Cluj-Napoca, Romania; 3. Nursing Department, Iuliu Hatieganu University of Medicine and Pharmacy, Cluj-Napoca, Romania

**Keywords:** dyspepsia, Helicobacter pylori, prevalence, respiratory test, students

## Abstract

Recent data suggest that the prevalence of Helicobacter pylori (HP) infection in Romania has been declining in the last 30 years. However, there are no studies regarding HP prevalence among medical students. The objectives of this study were to estimate the prevalence of HP infection and assess the prevalence of dyspepsia in medical students and the relationship between dyspepsia and infection. We included 150 students from the Iuliu Hatieganu University of Medicine and Pharmacy of Cluj-Napoca, Romania (102 females and 48 males, mean age 21 years). Each student completed a lifestyle questionnaire, personal history, family history as well as the Rome IV questionnaire for functional dyspepsia. The status of HP infection was determined using the C13-urea respiratory test. The prevalence of HP infection was 25.33%, and 18% met the Rome IV criteria for functional dyspepsia. 37% of students with functional dyspepsia had a positive HP test. Of all students, 8% had a history of HP infection. Those with a history of HP infection had a 4.45% (95% CI 1.6 – 12.37) higher risk of having positive Rome IV criteria for functional dyspepsia than those with no previous history of infection (p=0.008). Thus, the present study adds to the body of evidence regarding HP prevalence among medical students, 25.33% being positive. We found no statistically significant correlation between HP infection and functional dyspepsia. Those with a history of HP infection had a higher risk of functional dyspepsia.

## Introduction

Helicobacter pylori (HP) infection is one of the world’s most common infections [1] with many routes of transmission: direct contact between subjects [2], contaminated water, sources or food [3–5], zoonotic transmission, and iatrogenic transmission during endoscopies and dental care [6].

In 2019, a review that summarizes recent publications on the epidemiology of HP was published [7]. A continuous decrease in HP prevalence was reported from many regions worldwide, including Korea, China, Iran, and Austria. In these countries, the prevalence of the infection ranged between 27.5% in East China and 41% in the United Arab Emirates. Brazil already has a high prevalence of infection in young people, which has been estimated to be around 50% in children and 70–90% in adolescents and adults. In Ethiopia, a high overall pooled HP prevalence was identified (52.2%), although a trend of decreasing incidence has been observed over time. In contrast, regarding Europe, a high seroprevalence (82%) was reported in Spain. This review revealed many risk factors for this infection. First of all, there seems to be a higher risk of infection among health professionals, in particular those working in gastrointestinal units and dentistry, and also in other professions such as agriculture, forestry, and fisheries, as well as sewage workers, miners, and workers at institutions for the intellectually disabled.

There is a higher risk for acquiring the infection for those who consume raw water, uncooked seafood as well as uncooked vegetables. No association was found between the smoking status, smoking duration or intensity, and HP. Gastroesophageal reflux and sexual partners have been associated with a higher risk for HP acquisition, and gut microbiota was suggested to play a role in the intrafamilial transmission of HP. A modeling exercise for evaluating the potential risk of acquiring HP infection was conducted in Mexico and Japan, which demonstrated a continuous decline in the prevalence, explained by diminished transmission [8, 9].

This year, a study published by Loor *et al.* revealed that there had been a downward slope of HP infection in the last 30 years in Romania [10]. It seems the prevalence among young patients (aged between 20–30 years) decreased from 78.1% in 1994 to 65.3% in 2003 to 38.8% in 2018 and 10.6% in 2019. Of course, the diagnostic methods and the studied population were different. Although there are many ways of detecting this infection, the latest guidelines claim that the 13C-urea breath test is the best approach to the diagnosis of HP, with high sensitivity and specificity and excellent performances [11].

The HP infection prevalence among the medical staff is also important, given the risk of transmission from patients, mainly during interventions like endoscopies. Indeed, this supposition was confirmed in the case of gastroenterologists and depended on their length of activity [12]. A systematic review from 2018 in which all publications published during 1989 and 2015 related to HP infection prevalence in various occupations were analyzed concluded that HP infection is an occupational work-related disease with the highest prevalence among gastroenterologists who practice endoscopy (82%) and dentists (70%). Among medical students, it seems that the percentage of infection was small (6.4%) [13]. In Romania, no study has been performed regarding the study of HP infection prevalence amongst medical students.

The specific objectives of this study were: i) to estimate the prevalence of HP infection among medical students; ii) to assess the prevalence of dyspepsia in medical students and the relationship between dyspepsia and infection; iii) to determine the association of HP infection with potential risk factors such as age, sex, lifestyle and the area of residence.

## Material and Methods

### Study design and settings

A prospective study was performed on 150 first and third-year medical students from Iuliu Hatieganu University of Medicine and Pharmacy, Cluj-Napoca, Romania, for two months by the end of 2019, before the onset of the COVID-19 pandemic.

### Participants

Whether or not they had digestive symptoms, medical students that volunteered and signed the informed consent were tested for HP infection using the C13-urea respiratory test. We excluded from the study those who were not fasting or who had smoked 24 hours before testing or followed previous treatments with antiacids, proton pump inhibitors (PPI), or antibiotics (4 weeks prior to the test), those who had a history of HP infection for which they underwent the eradication protocol less than 8 weeks before our study and, of course, those who did not want to participate.

### Study protocol

All participants completed the informed consent, the Rome IV questionnaire for functional dyspepsia, and another ad-hoc questionnaire regarding demographic data, lifestyle, history of HP infection, previous treatments, alarm symptoms, and family history of gastric cancers.

For our study, we used the Helicobacter Test INFAI® kit. Each student gave two basal respiratory samples. Later, they ingested the solution with citric acid (1 gram of citric acid diluted in 200 ml of water); after, they drank the C13-urea from the bottle dissolved in 30 ml of water. After 30 minutes, two other respiratory samples were collected. Each sample was labeled with barcodes to ensure safe and distinctive identification during analysis. All participants were instructed to take a deep breath, hold for approximately 10 seconds, and then blow into a balloon until it was firm. All respiratory samples were collected according to the manufacturer’s instructions. The proportion of 13CO_2_ in the breath samples was determined by nondispersive infrared spectrometry and resulted as an absolute difference (Δ-value) between 0-minute and 30 minute-values. The cut-off point for discriminating HP negative and positive patients was determined to be Δ-value of 4‰ which means that an increase of the Δ-value more than 4‰ between basal and after urea ingestion respiratory samples indicated an infection.

### Statistical analyses

Students’ characteristics were presented as counts and percentages in the case of categorical data or as means and standard deviations in the case of normally distributed continuous data. Comparisons between two independent groups were performed with Chi-squared or Fisher’s exact test, in the case of categorical data, or with a t-test for independent samples in the case of the normally distributed continuous data. To quantify the importance of the relation between two binary variables, we used risk difference and relative risk along with a 95% confidence interval. A level of 0.05 was used for significance, and the two-tailed p-values were used for all statistical tests. All statistical analyses were carried out using the R environment software for statistical computing and graphics (R Foundation for Statistical Computing, Vienna, Austria), version 4.0.2.

## Results

### Demographic characteristics of the study population

Of all 150 students, 68% (102) were female, and 32% (48) were male. In terms of the environment of origin, 16.67% of them were from rural areas (Table 1). Most of the participants were from Transylvania (Western Romania); only 16% (24) of them were from other regions of Romania. The mean age of the group was 21, ranging from 18 years until 27 years. Forty-five were first-year students, and the rest of them, 105, were third-year students. The patient’s characteristics and the prevalence are shown in Table 1.

**Table 1.** The prevalence of infection and the patient's characteristics.

**Test**	**All (n=150)**	**Positive (n=38)**	**Negative (n=112)**	**P-value**
**Age (years), mean (SD)**	21.1 (1.37)	21 (1.25)	21.12 (1.42)	0.629
**Age groups (years), n (%)**				0.52
Group (1^st^ year) 18–20 years	45	13 (28.9)	32 (71.11)	
Group (3^rd^ year) 21–27 years	105	25 (23.81)	80 (76.19)	
**Gender**				0.122
Female, n (%)	102 (68)	22 (21.57)	80 (78.43)	
Male, n (%)	48 (32)	16 (33.33)	32 (66.66)	
**Place of residence**				0.179
Urban, n (%)	12(83.33)	29 (23.2)	96 (76.8)	
Rural, n (%)	25(16.67)	9 (36)	16 (64)	

SD – standard deviation.

### Data about the prevalence of HP, functional dyspepsia, and history of HP infection

The prevalence of HP infection was 25.33%, meaning that 38 of 150 students tested positive. According to the Roma IV questionnaire, 18% (27) met the criteria for functional dyspepsia. Of those, 27 students who met the criteria for functional dyspepsia, 37% (10) had a positive test. According to the questionnaires, 8% (12 of 150) had a history of HP infection. Six followed the eradication protocol with amoxicillin and clarithromycin, one of them had a treatment with levofloxacin, and 5 of them did not know what treatment they had followed. Of 12 students with HP history, 5 of them had a new positive test.

### Data on lifestyle, NSAIDs consumption, alarms symptoms, and family history of gastric cancer

Regarding alcohol consumption, 23.3% of the students did not drink at all, 67% occasionally, 8.6% at least 2 times/week, and 0.67% daily. Regarding smoking habits, 19.33% of the students from our study smoked. Regarding non-steroidal anti-inflammatory drugs (NSAIDS) consumption, 23.3% did not take NSAIDs at all, 8.67% used them weekly, and 68% very rarely. According to the questionnaire, 3 students had alarm symptoms: 2 had a history of rectal bleeding, and 1 had unexplained weight loss. Of all subjects, 9.33% (14 students) had a family history of gastric cancer. We found no statistically significant association between NSAID consumption, alcohol, smoking, and functional dyspepsia.

### HP test results contingent upon the year of study

In the study, first-year students had a 1.21 (95% CI 0.68 – 2.15) higher risk of HP infection (23.8%, n=25) than third-year students (23.8%, n=13). However, the association was not statistically significant (p=0.512).

### Association between the history of HP infection, HP test results and Rome IV criteria for functional dyspepsia

The association between functional dyspepsia and the history of HP infection was tested, and a p-value of 0.008 was obtained. Those with a history of HP infection had a 4.45% (95% CI 1.6 – 12.37) higher risk of having functional dyspepsia than those with no previous infection history. Those with functional dyspepsia had a 1.63 (95% CI 0.9 – 2.93) higher risk of HP infection than those without dyspepsia. The association was not statistically significant (p=0.123).

## Discussion

There are no Romania data concerning functional or uninvestigated dyspepsia among medical students. In our study, 18% met the criteria of functional dyspepsia according to the Rome IV questionnaire. However, we found no statistical association between dyspepsia and HP infection or between dyspepsia and lifestyle habits. The results of a study from South America conducted in 2014 revealed a prevalence of functional dyspepsia of 24% among 1923 medical students, dyspepsia being related to several behavior variables [14]. A higher percentage of dyspepsia, 43%, was reported among 176 preclinical medical students from the United Arab Emirates [15]. More recent data from 2018, also from South America, showed that from 1241 medical students, 46% of them had uninvestigated dyspepsia [16]. In the same year, another similar study conducted on 600 medical students presented that a quarter of them had gastroesophageal reflux disease (GERD), and almost 40% of them also had associated dyspeptic symptoms [17].

Given our results, we can see that there is no statistical difference between HP prevalence in first-year students compared to the prevalence found in third-year students. We chose to include in the study first-year and third-year students because they had internships in the Department of Internal Medicine, where the study was conducted at that time.

A cross-sectional study was conducted on 472 medical students and residents from a university in Brazil, the seroprevalence of HP being 31.4%, its value increasing from the basic level to residency (ranging from 23.4% in students up to 38.6% in residents), suggesting that contact with patients during clinical practice may constitute a risk factor for acquiring HP infection [18]. In another endemic area of Bangladesh, a study of 324 medical students for whom IgG anti-HP antibodies were tested revealed a high prevalence of infection: 50% for first-year students with an increased percentage (59%) for final-year students [19]. However, an Australian study did not find differences between first and fifth-year dental students and the general population [20].

Different studies from around the world regarding the prevalence of HP infection among medical students evidenced different values. In 2003, a study in Austria revealed that out of 242 medical students who were tested with the C13-urea breath test, 10% were positive. Similar to our study, there was no significant difference in HP prevalence between dyspeptic students and asymptomatic students [21]. In 2005, in a high endemic area from Saudi Arabia, out of 120 medical students, for which the C13-urea breath test was performed, the prevalence of HP infection was 35% [22]. In 2014, in Iran, out of 363 medical students tested for HP infection using the IgG serology, almost half of them (45.7%) had a positive test [23]. In Japan, the prevalence was lower: out of 449 students from medical school who were investigated using the rapid urease test or urine antibody, 7.3% were infected [24].

The results from a study conducted in Japan with a large but younger (high school students aged 12–16 years) number of patients concluded that out of 1298 subjects who were screened for HP infection using IgG anti-HP antibodies, only 3.2% were positive [25]. A review regarding the prevalence and management of HP infection among international students who came to the United States (US) was published in 2015. The prevalence of HP infection was high, ranging from 60% to 80% among the nations that send the most students to the US (mostly Asian nations), compared to the prevalence among American students, which was around 25% [26].

Other similar studies conducted on non-medical students from Europe showed a similar prevalence: in 2003, in Ireland, out of 170 students tested for HP using IgG anti-HP antibodies, 52% of them had a positive test [27]. In 2008, in Turkey, out of 200 students who were tested for HP infection by the monoclonal stool antigen test, a high percentage of them also had a positive test (63%) [28]. In Japan, all studies show a smaller prevalence of HP infection; out of 777 students tested with IgG anti-HP antibodies, 14.7% were positive [29]. The lower rate of HP infection and the gastric cancer rate in this area could be explained by the fact that in Japan, HP infection has been considered an indication for eradication since 2010, irrespective of background diseases, and this is approved by the Japanese health insurance system [30].

This epidemiological trend of HP infection among students from different countries is presented in Table 2, and as observed, there is a wide dispersion of the values of the prevalence of HP infection. In our study, from 150 students, 12 reported a history of HP infection for which they had treatment, and now 5 of them had a new positive result. This fact could be explained by inadequate bacterial eradication protocol or HP reinfection. In 2018, a meta-analysis that provides a comprehensive overview of the global antibiotic resistance of HP in the last ten years was published. The presented results showed worrisome levels of resistance rates in many areas of the world. In European countries, the primary resistance to clarithromycin was 18%, 11% to levofloxacin, and 32% fto metronidazole [31]. In 2020, a study detected a moderately high genetic HP resistance to antibiotics (clarithromycin and levofloxacin)

**Table 2.** Epidemiological trends of HP prevalence in students from different parts of the world.

**Reported year**	**Place**	**Population age**	**Number of subjects**	**Methodology**	**Prevalence**	**Reference**
**2003**	Austria	21–39 years	242 medical students	C14-Ureea breath test	10%	[21]
**2003**	Brazil	17–48 years	472 medical students and residents	IgG-anti-HP	31.4%	[18]
**2004**	Ireland	18–22 years	170 students	IgG-anti-HP	52%	[27]
**2005**	Saudi Arabia	18–28 years	120 students	C14-Ureea breath test	35%	[22]
**2007**	Japan	18–25 years	777 students	IgG anti-HP	14.7%	[29]
**2008**	Turkey	18–23 years	200 students	Ag stool test	63%	[28]
**2010**	Bangladesh	18–24 years	324 medical students	IgG anti-HP	50%	[19]
**2011**	Russia	18–30 years	594 students	Electrochemical breath test	60.4%	[33]
**2017**	Iran	18–24 years	363 medical students	IgG-anti HP	45.7%	[23]
**2018**	Japan	12–16 years	1298 junior high-school	IgG-anti HP	3.2%	[25]
**2019**	Japan	21–29 years	449 medical students	Rapid urease test and urine antibody	7.3%	[24]
**2020**	Romania	18-27 years	150 medical students	C14-Ureea breath test	25.3%	Our study

in the southeastern region of Romania [32]. An interesting study from Moscow was published in 2011: 594 first-year students were tested for HP infection using the electrochemical breath test and IgG anti-HP antibodies. The prevalence of the infection was high: 60.4%. Those with a positive test received the eradication protocol with amoxicillin, clarithromycin, and PPI; in 90% of the cases, the treatment was successful (the students were retested to ensure the eradication). Surprisingly, over time (in the fourth year), the HP infection rate for the same students was higher: 77.3% [33].

Nowadays, there are consensus and clinical guidelines regarding infection management at a European level and most European countries. However, no data have shown the level of implementation of these recommendations. Nevertheless, our study had its limitations: the relatively small number of subjects included in the study and the fact that we did not have other paraclinical examinations for those who were tested that could explain dyspepsia (e.g., parasitological stool examination, abdominal ultrasound etc). It would also have been useful to monitor the eradication rate of those with positive tests.

## Conclusion

To our knowledge, this is the first report on the prevalence of HP infection among medical students investigated with the C13 breath test in Western Romania. The prevalence was 25.33%. 18% of the students met the Rome IV criteria for functional dyspepsia. We found no statistically significant association between HP infection and Rome IV criteria for functional dyspepsia. Those with a history of HP infection presented a 4.45% (95% CI 1.6 – 12.37) higher risk of having functional dyspepsia..

## Acknowledgments

### Ethical approval

The ethical approval for this study was obtained from the Ethics Committee of the Iuliu Hatieganu University of Medicine and Pharmacy (approval ID: 471/19 December 2018).

### Consent to participate

Written informed consent was obtained from the participants.

### Conflict of interest

The authors declare that there is no conflict of interest.
